# Protocol to assess metabolic activity of *Pseudomonas aeruginosa* by measuring heat flow using isothermal calorimetry

**DOI:** 10.1016/j.xpro.2023.102269

**Published:** 2023-05-01

**Authors:** Katrin Beilharz, Kasper Nørskov Kragh, Blaine Fritz, Julius B. Kirkegaard, Tim Tolker-Nielsen, Thomas Bjarnsholt, Mads Lichtenberg

**Affiliations:** 1 Symcel Sweden AB, Tomtebodavägen 6, 171 65 Solna, Sweden; 2 Costerton Biofilm Center, Department of Immunology and Microbiology, University of Copenhagen, 2200 Copenhagen, Denmark; 3 Niels Bohr Institute, University of Copenhagen, 2100 Copenhagen, Denmark; 4 Department of Clinical Microbiology, Copenhagen University Hospital, Rigshospitalet, Copenhagen, Denmark

**Keywords:** Cell-based Assays, Metabolism, Microbiology

## Abstract

Here, we present a protocol for assessing metabolic activity of bacterial populations by measuring heat flow using isothermal calorimetry. We outline the steps for preparing the different growth models of *Pseudomonas aeruginosa* and performing continuous metabolic activity measurements in the calScreener. We detail simple principal component analysis to differentiate between metabolic states of different populations and probabilistic logistic classification to assess resemblance to wild-type bacteria. This protocol for fine-scale metabolic measurement can aid in understanding microbial physiology.

For complete details on the use and execution of this protocol, please refer to Lichtenberg et al. (2022).^1^

## Highlights

• Isothermal microcalorimetry (IMC) for fine-scale metabolic measurements
• Non-invasive metabolic measurements demonstrated for different bacterial growth models
• Novel data analysis approach distinguishes microbial populations based on thermogram

## Abstract (editor highlights)

Publisher’s note: Undertaking any experimental protocol requires adherence to local institutional guidelines for laboratory safety and ethics.

## Graphical abstract

**Figure:**
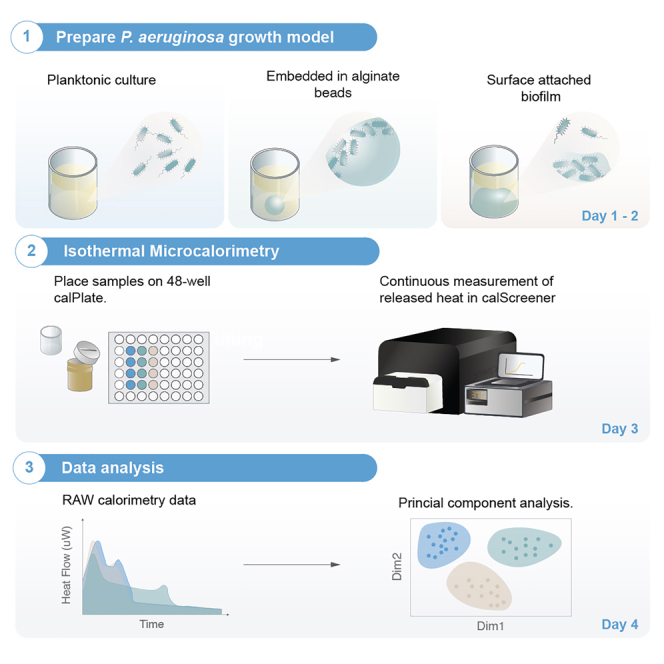

## Before you begin

Differences in growth geometry of bacteria depending on growth models, e.g., biofilms, cell aggregates or planktonic cultures, may impact their microenvironment and consequently the metabolic activity and degree of aggregation. Increased tolerance to antibiotics of biofilms, persister cells, spores and dormant cells have been correlated with lower metabolic activity of the bacteria.^2^^,^^3^

Non-invasive measurements of metabolic activity in live cells are challenging and most methods only measure isolated factors of the metabolic output, such as metabolites, oxygen consumption, or acidification. By analyzing metabolic activity in living cells, it is possible to identify energetically demanding processes and to distinguish different growth stages of bacteria.

Here we describe a calorimetry method in biological context (biocalorimetry) using the calScreener™ (Symcel AB, Sweden) for multiplexed, label-free, and direct population-level measurement of metabolic activity in live samples. Early protocols have been established for measuring a variety of bacterial species and mammalian cell types.^4^

The transition of planktonic to biofilm phenotype as well as the production of extracellular matrix components is regulated by second messenger molecule cyclic diguanylate (c-di-GMP).^5^^,^^6^^,^^7^ In planktonic and biofilm embedded bacteria metabolic levels are distinct, however, it is unknown how c-di-GMP affects the signaling. Fine scale measurements of released energy of c-di-GMP mutants in different growth models can elucidate the impact of c-di-GMP signaling on the metabolic rate.

Here, we have established a protocol to measure and analyze metabolic activity in different growth models for strains of *Pseudomonas aeruginosa* with varying internal levels of (c-di-GMP) as an example.^1^ However, the microcalorimetry and data analysis part of the protocol can be used for other strains and growth models.

Isothermal calorimetry measures dissipated energy from biochemical reactions within cells by monitoring released heat, a direct measure of metabolic activity. The measurement is thus unspecific to a certain process. Providing information on the total enthalpy changes over time (heat flow), which are not only related to biomass formation, but also physiological changes.^8^

The output data, a thermogram, displays heat flow (metabolic rate, μW) over time. Thermograms of different strains can be grouped by principal component analysis to detect differences in metabolic states of samples and further classified with logistic regression. Moreover, the non-invasive nature of the method allows for subsequent experiments on the same sample.

***Note:*** The protocol below describes the specific steps for setting up different growth models of *Pseudomonas aeruginosa* for planktonic, surface attached biofilm and alginate-beads embedded cells. Furthermore, we present a sensitive method for continuously measuring the metabolic activity of these models by biocalorimetry using the calScreener™ (Symcel AB, Sweden). For analysis, raw heat-flow data are used for principal component analysis (PCA) and logistic regression to distinguish the different metabolic states.

***Note:*** Here we will describe the method using lysogeny broth (LB) as growth media due to its ease of access and universal use in microbiology, but we have also used other growth media like R2A, tryptic soy broth (TSB) etc.

### Prepare bacterial culture

**Timing: 1 day**

The steps below are prepared one day prior to experimental setup.

1. Prepare overnight (ON) cultures of strains of interest. Here we describe *Pseudomonas aeruginosa* (WT, PA1120, and PA2133)*.*
2. Transfer cells from −80°C glycerol stock with an inoculation loop into 5 mL sterile LB in a 15 mL cell culture tube with dual position lid to allow air entering the tube.
3. Incubate for 24 h at 37°C on a shaking platform at 180 rpm.

### Sterilize calorimetry equipment

**Timing: 2 h**

4. Sterilize all equipment that is not single-use and already sterile by autoclaving.

5. Prepare calPlate™ and calWells™
  a. Sterilize titanium vials and lids by heat treatment in oven (160°C > 2 h). Alternatively sterilize by autoclaving, ethanol 70% (minimum 30 s) or UV light (minimum 30 min).
  b. Keep in a sterile box until usage.
    ***Note:*** Keep vials dry! Condensation, evaporation or residual liquid can interfere with the signal.
  c. Place all 48 titanium vials in the metal holder (calPlate™).

## Key resources table

**Table:** 

REAGENT or RESOURCE	SOURCE	IDENTIFIER
**Bacterial strains**		

*Pseudomonas aeruginosa* strain PAO1 WT	Pseudomonas Genetic Stock Center at East Carolina University	PAO10001
*PAO1 ctx::pBAD1120 pENTRCTX::pBAD-PA1120,* (PA1120) *encodes a diguanylate cyclase that synthesizes c-di-GMP. Inducible with arabinose.*	Rybtke et al.^9^	N/A
*PAO1 ctx::pBAD2133 pENTRCTX::pBAD-PA2133,* (PA2133) *encodes a phosphodiesterase that degrades c-di-GMP. Inducible with arabinose*	Andersen et al.^10^	N/A

**Chemicals, peptides, and recombinant proteins**		

LB broth	Sigma USA	Item no L3522
L-Arabinose	Sigma, USA	CAS no 5328-37-0
Seaweed alginate. Protanal LF 10/60 FT	FMC Biopolymer, Norway	N/A
CaCl_2_	Merck, USA	CAS no 10035-04-8
NaCl	Merck, USA	CAS no 7647-14-5
Glycerol	Sigma, US	CAS no 56-81-5

**Software and algorithms**		

calView v 1.033	Symcel AB, Sweden	N/A
Python v. 3.9.1	Python Software Foundation	N/A
numPy v. 1.20.2	Python Software Foundation	N/A
Matplotlib v. 3.3.4	Python Software Foundation	N/A
scikit-learn v. 0.24.1	Python Software Foundation	N/A
python-ternary v. 1.0.8	Python Software Foundation	N/A
PCA analysis and logistic regression classification	Zenodo.org	https://doi.org/10.5281/zenodo.7541038

**Other**		

calScreener™	Symcel AB, Sweden	1200001
calWells (inserted in 48-well titanium calPlate)	Symcel AB, Sweden	1220089/-91
calPlate	Symcel AB, Sweden	1220093
calData - web-based analysis tool	Symcel AB, Sweden	https://symcel.com/analysis-tools/calorimetric-growth/

## Materials and equipment

In this protocol, metabolic activity is measured in up to 32 samples simultaneously using a calScreener™ (Symcel AB, Sweden) biocalorimeter. For production of alginate beads, surface biofilms, and planktonic culture, prepare the following solutions prior to experiments.

• **2% alginate solution:** Add 2 g of Sodium alginate (Protanal LF 10/60 FT) to 98 mL milliQ water under stirring on a heated magnetic stirrer set to 500 rpm and 100°C and allow to completely dissolve (∼30 min).

Autoclave and store at 4°C. Use within one month and ensure that solution is sterile before use.

• **0.25 M CaCl**_**2**_**solution:** Add 36.755 g CaCl_2_ (dihydrate) in 1,000 mL milliQ water.

Autoclave and store at 4°C up to 36 months.

• **20% L-arabinose:** Add 2 g of L-arabinose to 8 mL milliQ water and sterile filter through a 0.22 μm syringe filter.

Store at 4°C. Use within one week.

• **0.9% saline solution:** Add 9 g NaCl to 100 mL milliQ.

Filter sterilize and store at +4°C for up to 24 months.

• **LB broth:** Weigh 25 g LB powder and suspend in 1,000 mL milliQ water and mix until everything is dissolved.

Autoclave and store at 4°C. Use within one month and ensure that medium is sterile before use.

## Step-by-step method details

### Preparation of growth models: surface attached biofilm (SB), alginate bead embedded cells (AB) and planktonic cultures (PC)

**Timing: 2 days**

This section describes the preparation of *P. aeruginosa* growth models for surface attached biofilm, alginate bead embedded cells and planktonic cells. Surface biofilms is incubated directly inside non-activated plastic inserts (calWells™, Symcel AB, Sweden) to allow further metabolic measurements, while alginate beads are incubated in Erlenmeyer flasks and planktonic cultures in cell culture tubes and then transferred to plastic wells upon measurements.

Surface attached biofilm formation has been optimized for the plastic inserts and the planktonic culture preparation has been adapted. The encapsulation of bacteria in alginate beads is done using a modification of previously described protocols.^11^^,^^12^

**Figure 1:**
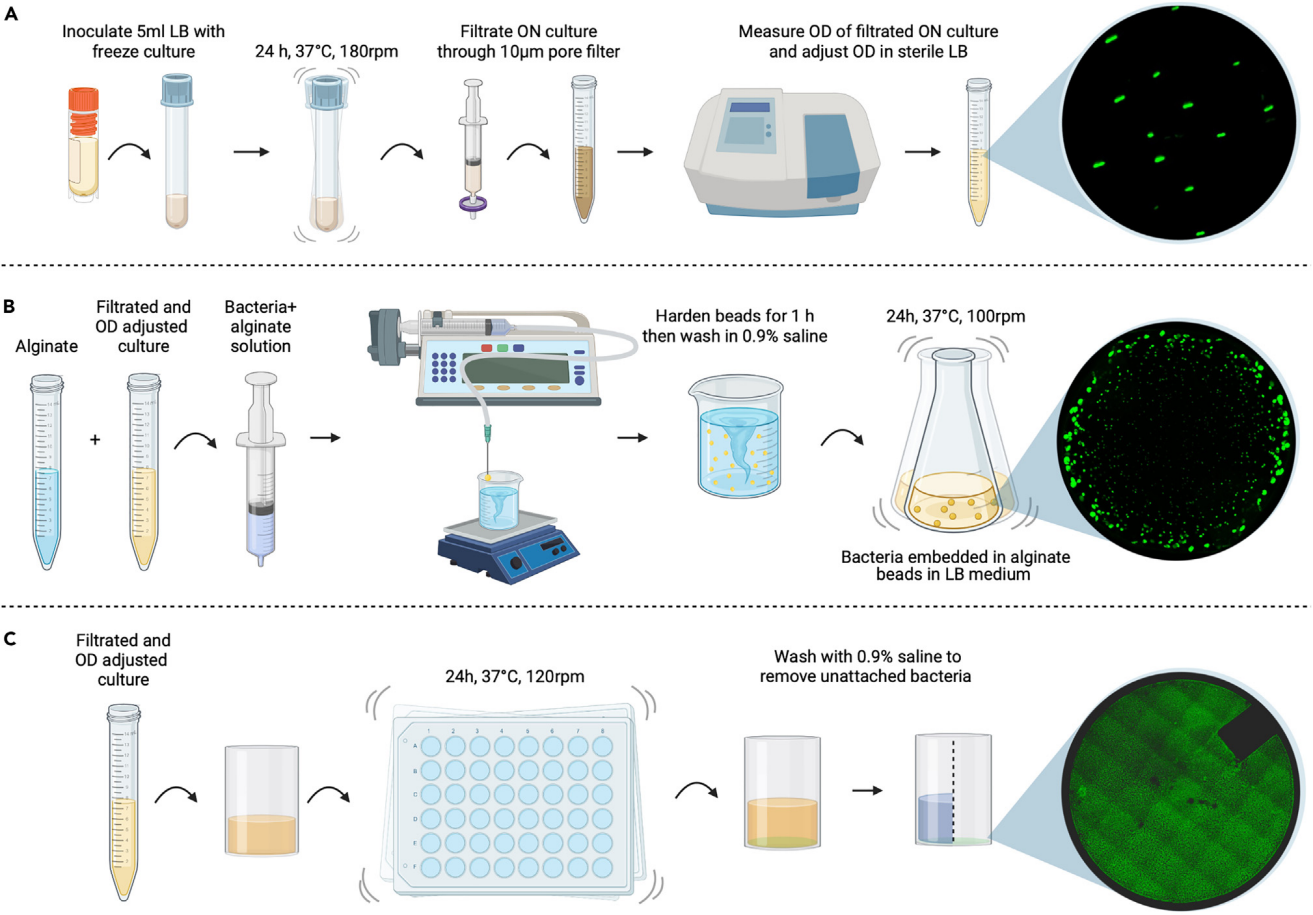
Workflow of preparing *Pseudomonas aeruginosa* in different growth models (A) For planktonic cultures, 5 mL LB is inoculated from −80°C freeze stock and shaken at 180 rpm for 24 h at 37°C. The ON culture is then filtered through a 10 μm syringe filter and adjusted to an OD_600_ of 0.005 using a spectrophotometer. (B) For production of alginate beads, a filtered and OD_600_ adjusted ON culture is mixed with alginate. Following homogenization, alginate beads containing bacteria are produced by extrusion dropping into a stirred CaCl_2_ solution and beads are hardened for 1 h before being washed and transferred to Erlenmeyer flasks containing LB medium. (C) For production of surface biofilms, a filtered and OD_600_ adjusted ON culture is pipetted into individual plastic inserts, positioned in a standard 48 well plate and incubated for 24 h in 37°C and shaken at 120 rpm. Following incubation, media is removed, and the wells washed with sterile saline, and then transferred to titanium vials of the calPlate.

The full workflow is illustrated in Figure 1.

***Note:*** Two of the strains used here have an arabinose-inducible promoter. Thus, 0.2% L-arabinose (final concentration) should be added to induce the promoter. Here, we mixed LB with arabinose for inoculation into the calWells™ just prior to insertion in the calScreener™.

***Note:*** Use plastic inserts of calWells™ for setting up the growth model; that allows a direct measurement of the samples.

### Prepare a planktonic culture

**Timing: 1 h**

1. Filter the ON culture through a 10 μm syringe filter to ensure a mainly planktonic population.
2. Measure the OD of the filtered ON culture on a spectrophotometer and adjust to a final OD_600_ of 0.005 in sterile LB supplemented with 0.2% arabinose.
3. Add an aliquot of 200 μL to each plastic insert.
4. Position plastic inserts into titanium vial of the calPlate™ (Symcel AB, Sweden)

### Preparation of surface attached biofilm in plastic inserts

**Timing: 25 h (preparation ∼1 h; incubation ∼24 h)**

5. Filter the ON culture through a 10 μm syringe filter to ensure a mainly planktonic population.
6. Measure the OD of the filtered ON culture on a spectrophotometer and adjust to a final OD_600_ of 0.005 in sterile LB.
7. Fill the sterile plastic inserts (Symcel AB, Sweden) with 200 μL of the OD-adjusted ON culture.
8. Cover the plastic inserts with parafilm, position them in a 48-well plate and incubate for 24 h at 37°C, 120 rpm for biofilm to develop on the sides and bottom of the insert.
9. Following incubation, remove planktonic biomass by removing supernatant gently, then washing gently with 200 μL sterile saline solution (0.9% NaCl).
10. After washing, position inserts into titanium vials of the calPlate™ (Symcel AB, Sweden).
11. Add 200 μL fresh LB media supplemented with 0.2% L-arabinose to each plastic insert.

### Preparation of *P. aeruginosa* encapsulated in alginate beads

**Timing: 26 h (preparation ∼1.5 h; incubation ∼24 h)**

Encapsulation of *P. aeruginosa* in alginate beads follows the procedure as described elsewhere.^11^^,^^12^ In case of problems forming the spheres see troubleshooting 1.

12. Filter the ON culture through a 10 μm syringe filter to ensure a mainly planktonic population.
13. Measure the OD of the filtered ON culture on a spectrophotometer and adjust to an OD_600_ of 2.
14. Dilute the OD adjusted culture 20× in a sterile alginate solution (2% w/v) to a final OD_600_ of 0.1.
15. Place 150 mL of sterile 0.25 M CaCl_2_ in a 250 mL beaker glass on a magnetic stirrer with a sterile magnet (∼3 cm length).
16. Set the magnetic stirrer to 150 rpm and place a 21-gauge needle perpendicular to- and ∼3 cm from the surface of the CaCl_2_ solution, and ∼1.5 cm from the edge of the beaker.
17. Connect the needle to a 20 mL syringe containing the alginate-bacterial solution by sterile silicone tubing (internal diameter = 2 mm).
18. Mount the syringe into a syringe pump and set the flow rate to 30 mL/h.
19. Dispense droplets of the alginate-bacteria solution into the stirred CaCl_2_ solution.
20. Harden the spherical beads for 1 h in the 0.25 M CaCl_2_ solution.
21. Discard the CaCl_2_ and wash the beads twice by swirling the beads carefully in 50 mL sterile 0.9% saline solution in a beaker.
22. Transfer 20 beads to 50 mL prewarmed LB medium in a 250 mL Erlenmeyer flask.
23. Incubate at 37°C and 100 rpm for 24 h.
24. Transfer beads to a new beaker and rinse them gently by swirling in 50 mL 0.9% sterile NaCl for <1 min to remove non-embedded bacteria.
25. Place a single bead into a plastic insert (calWell™, Symcel AB).
26. Position plastic inserts into titanium vials of the calPlate™ (Symcel AB, Sweden).
27. Add 190 μL fresh LB medium supplemented with 0.2% L-arabinose resulting in 200 μL total volume in each well.

***Note:*** Handle the filled inserts carefully to avoid spilling. It is advised to use sterile tweezers for insert handling to prevent contamination. Liquid on the rim of the vial can interfere with the signal (Figure 8A).

### The calScreener biocalorimetry procedure

**Timing: 1–2 days (or longer)**

In this step, we set up the calPlate™ with the sample-containing plastic inserts and perform live cell metabolic measurements. Isothermal calorimetry with the calScreener™ allows evaluation of overall metabolic activity of a sample by continuous measuring of heat flow (HF, μW). Samples are prepared prior to insertion and measurement, which can be done for unlimited time, typically 24 h for fast growing cells.

We have optimized planktonic culture and a surface attached biofilm model for instrument specific plastic inserts, including cell number and pre-incubation times.

### Setup calPlate™ for measurement

**Timing: 1–2 days (or longer)**

**Figure 2:**
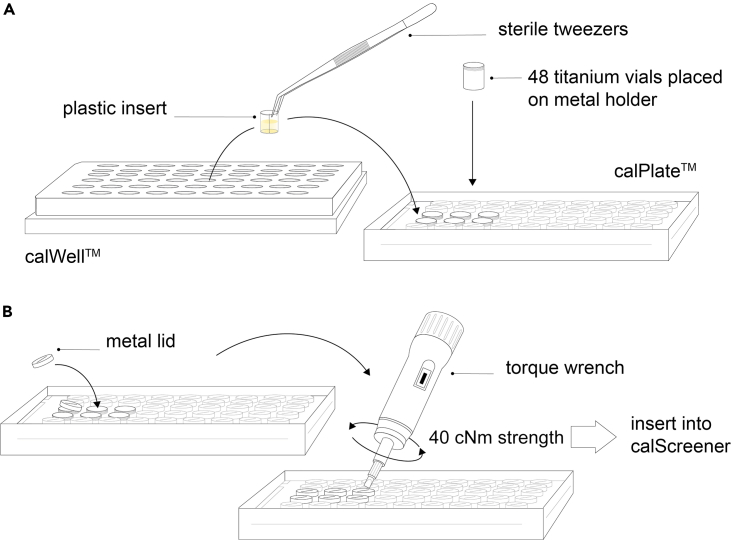
Schematic setup of the calPlate™ (A) After sample preparation, (A) transfer samples in plastic inserts into titanium vials that are placed in the 48 well plastic holder. (B) Place the lids on the titanium vials and close them with the help of a torque wrench.

Setup the calPlate™ (Figure 2) and insert into the calScreener™ for automatic and continuous measurement of released heat. Vials at positions A 1–8 and F 1–8 serve as thermodynamic references and contain sterile medium, that is used for the sampled. Culture and medium controls shall be added separately to vials at sample positions (B1–E8).

28. Place 48 sterile titanium vials into the metal holder of the calPlate™ on the sample station.
29. Place plastic inserts to vials in reference positions (A 1–8 and F 1–8).
30. Fill reference vials with equal volume and media as used for the sample vials, here 200 μL LB medium.

***Note:*** Use gloves and/or sterile tweezers to minimize the risk of contamination.

31. For biofilm and alginate bead samples, transfer the plastic inserts containing the samples in the titanium vials on the plate.
32. For planktonic cultures, place empty plastic inserts into titanium vials on the calPlate™ and subsequently add 200 μL aliquots of diluted samples to the vials.
33. Place the lids loosely on the vials (Figure 2B).
34. Tighten the lids using the torque wrench set on 40 cNm force.

### Measure metabolic activity of the samples

**Figure 3:**
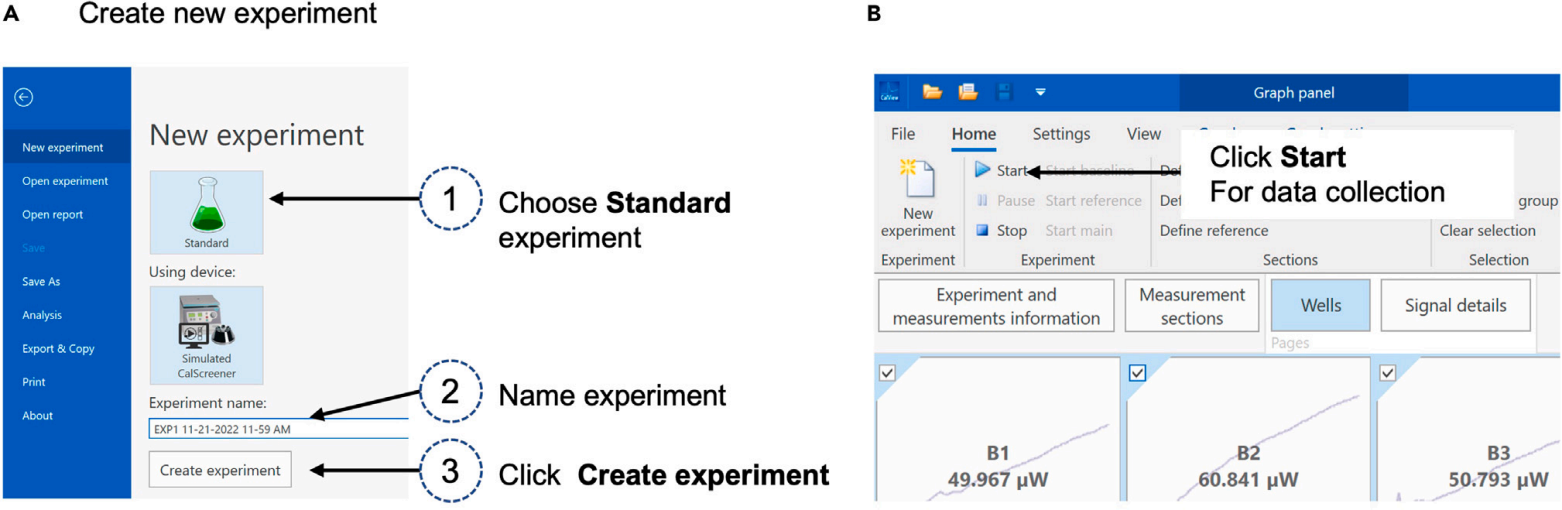
How to start a new measurement (A and B) In calView (A) create a new experiment and (B) start data collection prior to plate insertion.

**Figure 4:**
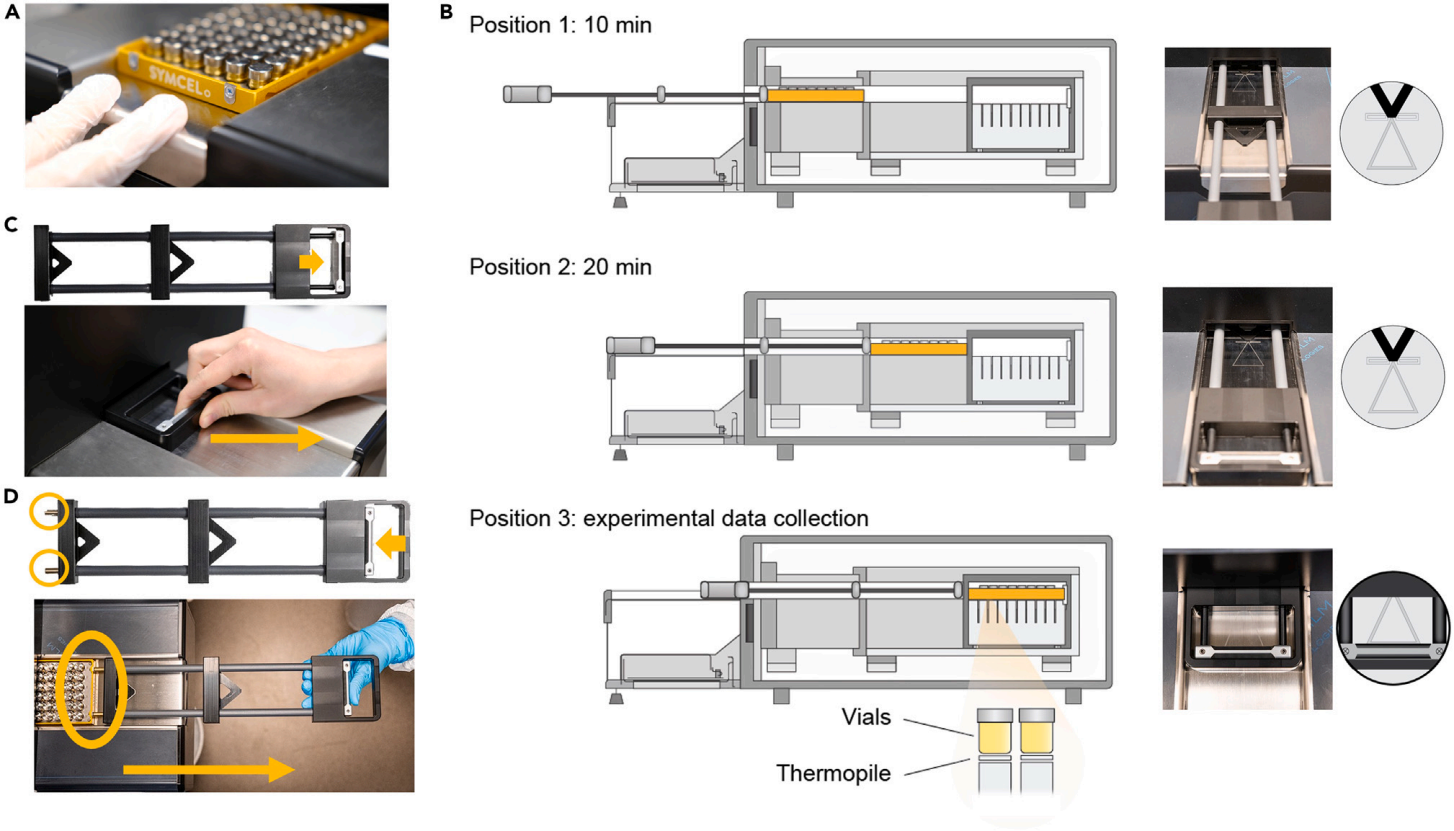
Plate handling (A) Place the calPlate in the correct orientation on the bridge. (B) Stepwise insertion, via position 1 (10 min) and position 2 (20 min), of the plate to allow temperature equilibration to instrument temperature, here 37°C. (C) Retract the plate handling arm with the magnets pulled in during measurement. (D) Retrieve the plate with help of the magnets of the plate handling arm.

35. Prepare the system for measurement.
  a. Check the temperature setting on the temperature control unit; it should be set at 37°C.
  b. Open calView™ software and start new experiment (Figure 3).

36. Stepwise insertion of the calPlate™ with temperature equilibration steps (Figure 4).
  a. Retract the plate insertion arm from the calScreener™.
  b. Place the calPlate™ on the bridge (column 8 facing insertion opening).
  c. Gently push the calPlate™ on position one using the plate insertion arm and let the system stabilize for 10 min.
    ***Optional:*** annotate sample content in software for individual vials.
  d. Push plate insertion arm to position two and wait for 20 min for temperature equilibration of the system.
  e. Push plate to final position 3 (measuring position) and activate **Reaction Start** in calView™.
  f. Retract plate insertion arm to Start position (Figure 4C).
  g. Run experiment for desired time (e.g., 24 h).
    ***Note:*** Check that the plate is correctly placed on the heat sensor. Troubleshooting 3 (Figure 7)
    **CRITICAL:** Make sure you do not move the plate from the sensor when retracting the arm.

37. Remove calPlate™.
  a. Press **Stop** in calView™ to end the experiment and save the file for further analysis.
  b. Insert the plate insertion arm completely and retrieve the calPlateTM with help of the magnets (Figure 4D).
  c. Open the lids using the torque and retrieve the sample for further analysis if required, otherwise discard.

### Validate data quality

**Timing: 1 h**

Check and validate quality of measurement and prepare data for analysis.

38. Open the experiment file in calView™.
39. Normalize data with baseline correction (select approx. 20 min in lag phase with signal between 0–10 μW).
40. Export ∗csv file (for analysis in calData, GraphPad, Microsoft Excel or any other compatible software solution).
41. Check data quality and see if there were any issues with the measurement; see troubleshooting 2, troubleshooting 4, troubleshooting 5 and troubleshooting 6.

**Figure 5:**
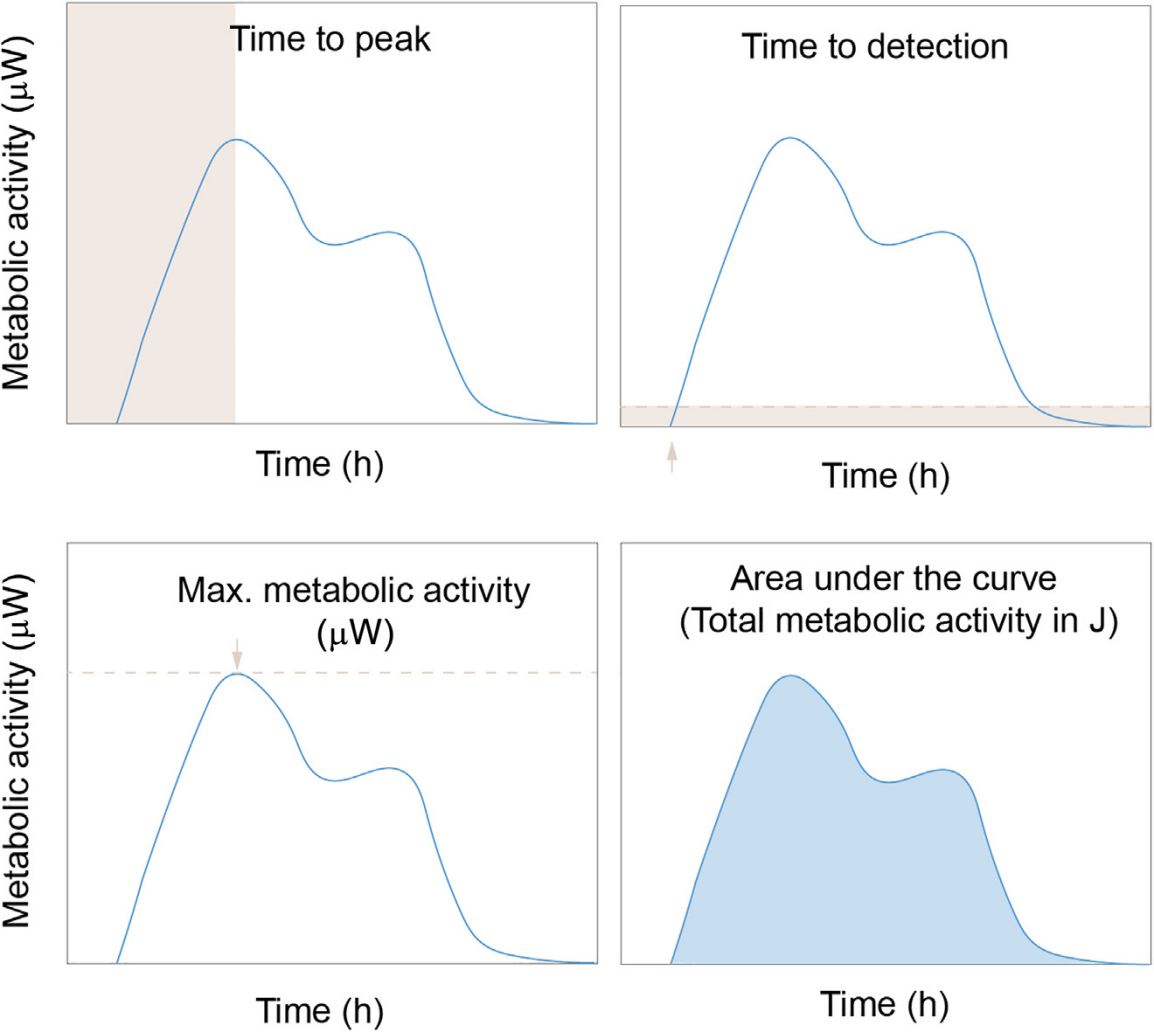
Metabolic parameters that can be analyzed with the help of calData Examples are time to the first signal maximum (Time to peak), time to signal detection, maximum metabolic activity (μW) and total metabolic activity (J). These parameters can be compared across different samples.

***Optional:*** For tailored metabolic parameter analysis upload data to webtool calData. https://symcel.com/analysis-tools/calorimetric-growth/ (Figure 5).

## Expected outcomes

**Figure 6:**
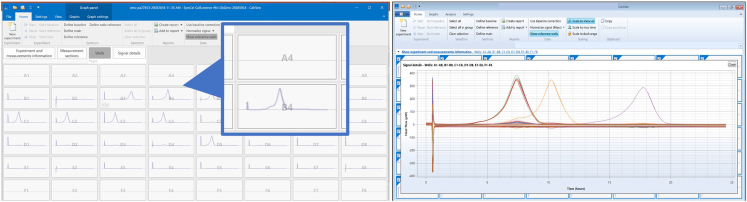
Expected outcome Example of good quality data from measurement of a bacterial sample. After the stepwise insertion of the plate, there will be an initial peak when the plate in finally loaded on the thermophiles (heat sensor). These are thermal disturbances deriving from small changes in temperature or frictional heat. This ceases quickly following an exponential decay function and establishing a stable measurement.

A metabolic profile (thermogram) for different growth models, as well as species and growth condition, shows differences in shape, regardless of preincubation time. An example for a thermogram is shown in Figure 6. After inserting the plate, the reference signal drops and the sample signal rises, stabilizing around zero when temperature equilibrium is reached. It depends on the initial inoculum how long the lag period is before reaching the detection limit. A positive heat flow indicates metabolic activity.

Alteration of internal c-di-GMP levels contribute to metabolic activity changes where for example high c-di-GMP levels lead to an increase of the maximum metabolic activity as shown for planktonic and biofilm samples.^1^

IMC allows for testing fast antibiotic susceptibility in real time, with dose dependent increase of time to signal detection.^13^^,^^14^ Depending on the compound other parameters, such as maximum metabolic rate of total heat, of the thermogram will be affected. Further it allows for real time monitoring of biofilm sensitivity, with a reduction of metabolic activity, and detection metabolic mutants that can effect antibiotic susceptibility in biofilms.^15^^,^^16^

## Quantification and statistical analysis

### Prerequisites

To run analysis code in Python you must install Python using either the official binary found at:

https://www.python.org/downloads/

or the Anaconda version found at:

https://www.anaconda.com/products/distribution.

Subsequently, required packages can be installed by running the command in a command prompt/terminal:

pip install numpy matplotlib scikit-learn python-ternary

or

conda install numpy matplotlib scikit-learn python-ternary

depending on whether Python was installed using anaconda or not.

### Data preparation

Collect all data in a comma-separated spreadsheet. We provide example data (data.csv) and example scripts that assume the following format:

***Note:*** Ensure that all columns have equal length or trim the data accordingly if this is not the case.

### Principal component analysis

To analyze the outcome of the experiments using principal component analysis, run the included script pca.py, which executes the following steps:

1. Gather the metabolic signal data in a single matrix of shape EXPERIMENTS × TIMESTEPS. See Table 1.

**Table 1:** Example for provided data set

Dataset	AB_High_1.1	AB_High_1.2	AB_High_1.3	AB_Low_1.1	⋯
Model	AB	AB	AB	AB	⋯
C-di-GMP	high	high	high	low	⋯

Time (h)					
0	33.12090005	30.7262476	33.43149837	25.61715736	⋯
0.016666667	31.91607604	29.1767515	31.86438634	24.81622905	⋯
0.033333333	31.10763502	28.16090497	30.80916211	24.28792922	⋯
0.05	30.5501568	27.46425527	30.06644761	23.95762386	⋯
⋮	⋮	⋮	⋮	⋮	⋱
2. Run PCA decomposition of the data using sklearn.decomposition.PCA.
3. Transform the data to the two principal components.
4. Plot the data as a labeled scatter plot.

### Wild-type classification

To classify the strains in terms of wild-type models, run the included script classification.py, which executes the following steps:

5. Select all wild-type data and create an input matrix of shape WT_EXPERIMENTS × TIMESTEPS of metabolic signal and a vector of length WT_EXPERIMENTS storing the type of model used in the experiment.
6. Train a probabilistic logistic classifier on the wild-type data.
7. Use the classifier to evaluate all probability of wild-type model on all data.
8. Plot the data, which, in the case of three classes, can be done efficiently as a ternary plot.

## Limitations

Biocalorimetry measures the thermal activity of a sample, a measure that is proportional to metabolic activity. The method is particularly useful for applications in microbiology and in this protocol, we describe the measurement of metabolic activity of different growth models. IMC is a robust and sensitive method, however, there are certain limitations to consider.

IMC measures heat released at the population level and sums all metabolic processes within a sample. Despite the high sensitivity of the measurement and its ability to detect minute changes, the non-specific nature of the signal poses certain challenges for interpretation.

In the case of surface biofilm, we must consider that not only biofilm embedded cells but also planktonic cells will contribute to the signal, and this cannot directly be distinguished from the signal. This is also the case for co-culture experiments of mixed species, where every single cell, therefore species, contributes to the overall signal. Although the thermograms are species and condition specific, the data can benefit from complementing experiments. Therefore, it is of advantage that the technique is non-invasive and non-disruptive which allows for endpoint assays on the same sample. Online measurements in a replicate sample are possible.

For comparison of principal component analysis of different samples, a determination of growth rate may be required when focusing on metabolic changes only. That way we can exclude growth rate effects on the metabolic signal.

During the measurements, samples are kept in a closed system. This will eventually lead to an oxygen depletion, acidification, and accumulation of metabolic byproducts may occur which has to be taken into consideration for analysis. Although, the design of the vials totaling 600 μL internal volume and working with volumes of 100–200 μL allow sufficient oxygen diffusion from the headspace of the vial for an extended amount of time.

The measurement can be performed for an unlimited time, especially when observing physiological rearrangements in stationary phase. However, to determine when to stop the experiment depends on the system one is working with. The possibility for online monitoring of metabolic activity is very useful to determine the experimental endpoint, for instance when the heat flow values of the samples decay and reach close to zero values.

To achieve maximum sensitivity and accuracy, the samples when introduced to the system must equilibrate to the assay temperature which takes approximately 45 min until data can be collected. In microbiology experiments, one typically starts with a low inoculum where the lag phase until signal detection exceeds equilibration time. For most experimental setups, this time lag is not a significant disadvantage, unless acute responses are being measured. Therefore, the method is applicable for a wide range of systems (organisms), including slow growing organisms such as for example *Mycobacteria, Cutibacterium acnes,* filamentous fungi etc.

For samples and experiments that require temperatures below 30°C the system cannot be directly applied. The calScreener™, has an active heating but not active cooling system and requires a temperature difference (delta T) of at least 10°C to provide a stable signal. For low temperature measurements an external climate chamber is required.

Instruments must be placed in a temperature stable environment, ideally a temperature-controlled room, to ensure accurate measurements for temperature fluctuations that can lead to baseline variability (See also troubleshooting section for noise).

## Troubleshooting

### Problem 1

Alginate beads are not spherical, or they are dissolving.

### Potential solution

The method for producing alginate beads is not the main focus of this protocol and has been extensively described elsewhere.^9^^,^^11^^,^^12^^,^^17^^,^^18^

### Problem 2

Thermograms show no or very short lag phase.

### Potential solution

• Setup a new experiment where samples are more dilute.
• To avoid such issue, run a range of dilutions to find optimal conditions.

### Problem 3

**Figure 7:**
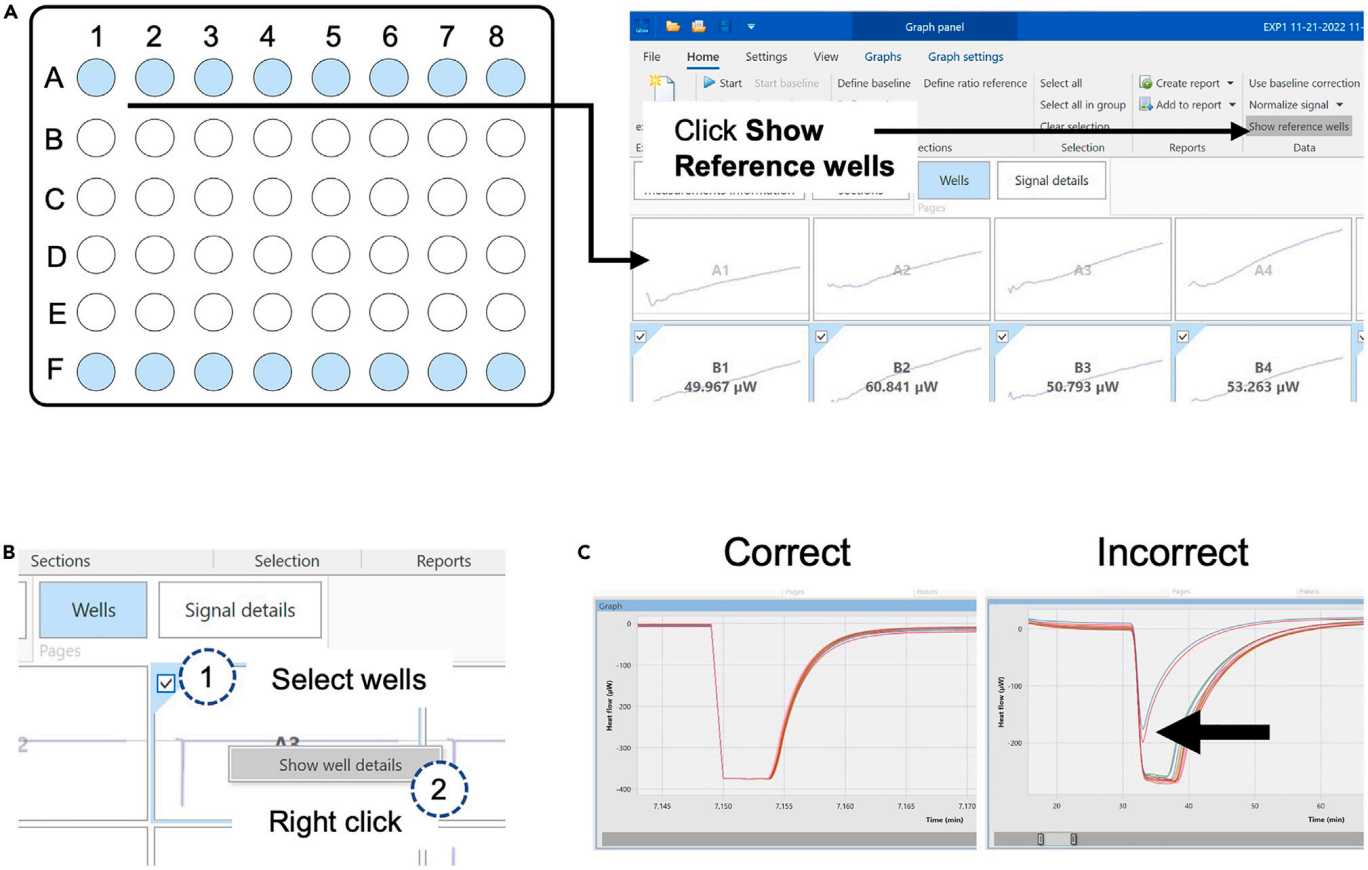
Check for correct plate insertion from the heat flow in the thermodynamic references (A and B) After plate insertion and start of the data recording (A) click on ‘Show reference wells’ in calView and (B) choose to show well details (heat flow). (C) Shown here are examples for signals for correct and incorrect plate insertion.

Vials are not correctly placed on the thermal sensors (Figure 7).

### Potential solution

• Check the thermograms of the thermodynamic reference (A 1–8 and F 1–8) after insertion of the plate.
• The thermograms should move to negative values of the heat flow (μW) and plateau for a couple of minutes before going up and stabilizing at zero value again (see Figure 7C).
• If the signal does not plateau, re-enter the plate directly and make sure that the plate is pushed all the way in.
• The insertion arm should be retracted and not touch the plate during the run.
• To avoid plate retraction, hold the silver handles when retracting the arm (see Figure 4C).

### Problem 4

**Figure 8:**
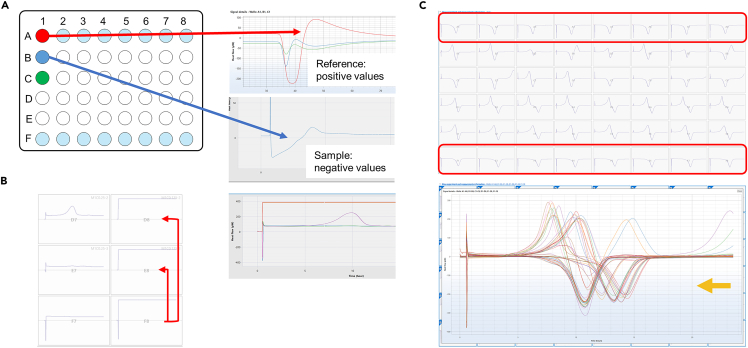
Examples for signal interference (A–C) Thermograms are shown for (A) moisture under the lid in reference or sample vial, (B) leakage from the lid in reference vial and (C) contamination in reference vial.

Negative initial signal due to leakage (Figure 8).

### Potential solution

• When lids are fully tightened, the titanium vials resemble a closed system. In case of leakage from the vial, condensation, an endothermic process, occurs. This leads to a negative initial signal that stays constant throughout the measurement (Figure 8B).
• Leakage can have several different sources:
  ○ Displaced or brittle O-rings.
  ○ Not fully tightened lid.
  ○ Liquid residual on O-ring.
• Retract the plate and check the lid of the vial where the signal disturbance occurred.
• If the lid was not fully tightened, tighten it again and reinsert the plate for measurement or start new experiment.
• If an O-ring is missing, displaced, or worn out it must be replaced.

***Note:*** It is advised to check the lids regularly to see that the O-rings are not damaged.

### Problem 5

Moisture under the lids (Figure 8A).

### Potential solution

• Moisture under the lid changes the signal in a particular way which can be recognized in the data (Figure 8A). Examples for moisture in a thermodynamic reference in A1 depicted in red (A1) or in a sample vial in blue (B2).
• Make sure to remove spills on the vials to avoid moisture under the lid.
• When transferring the vials to the plate or when carrying the plate, be careful to not move it too much.
• Before closing the lids, check if the O-ring is in place.
• Screw the lid on with the appropriate torque and do not use force.
• Before analyzing data, check for data quality.
• Discard data with signal interference from moisture and open lid.

### Problem 6

Contaminations in reference vials.

### Potential solution

• Thermodynamic references contain medium, here LB, and should give a steady signal. In case of contamination in reference vials, negative heat flow values occur, and it will impact referring sample vials.
• An example is shown in Figure 8C. Contaminated thermodynamic references (A4, F2-F8) change thermograms of samples that show negative values as indicated with yellow arrow.
• Check the thermogram of the reference wells (A 1–8 and F 1–8). The heat flow should plateau around zero throughout the course of the experiment.
• If the values move to negative values, that indicates microbial contamination of the reference and connected samples should be discarded.
• The references refer to the two lower samples in the same column, e.g., reference A1 to sample B1 and C1. Or to the reference row F, the two upper ones, e.g., D1 and E1 for reference F1.

### Problem 7

Noisy or fluctuating signal in the references.

### Potential solution

• Noise in the references could indicate that the room where the instrument is placed has temperature fluctuations.
• To find out whether that is the reason, measure the temperature over a longer time interval of a couple of days.

## Resource availability

### Lead contact

Further information and requests for resources and reagents should be directed to and will be fulfilled by the lead contact, Mads Lichtenberg (mlichtenberg@sund.ku.dk).

### Materials availability

Mutant strains generated for use in this study will be made available on request, but we may require a completed Materials Transfer Agreement if there is potential for commercial application.

### Data and code availability

All original code has been deposited at Zenodo and is publicly available as of the date of publication (https://doi.org/10.5281/zenodo.7541038).
